# Exploring the Prokaryotic Community Associated With the Rumen Ciliate Protozoa Population

**DOI:** 10.3389/fmicb.2018.02526

**Published:** 2018-10-29

**Authors:** Bar Levy, Elie Jami

**Affiliations:** ^1^Department of Ruminant Science, Institute of Animal Sciences, Agricultural Research Organization, Volcani Center, Rishon LeZion, Israel; ^2^The Mina and Everard Goodman Faculty of Life Sciences, Bar-Ilan University, Ramat Gan, Israel

**Keywords:** rumen, microbiome, endomicrobia, methanogenic archaea, symbionts, ciliate protozoa

## Abstract

Ciliate protozoa are an integral part of the rumen microbiome and were found to exert a large effect on the rumen ecosystem itself as well as their host animal physiology. Part of these effects have been attributed to their ability to harbor a diverse ecto- and endo-symbiotic community of prokaryotic cells. Studies on the relationship between the protozoa population and their associated prokaryotic community in the rumen mainly focused on the methanogens, revealing that protozoa play a major role in enhancing methanogenesis potential. In contrast, little is known about the composition and function of the bacteria associated with rumen protozoa and the extent of this association. In this study, we characterize the prokaryotic communities associated with different protozoa populations and compare their structure to the free-living prokaryotic population residing in the cow rumen. We show that the overall protozoa associated prokaryotic community structure differs significantly compared to the free-living community in terms of richness and composition. The methanogens proportion was significantly higher in all protozoa populations compared to the free-living fraction, while the Lachnospiraceae was the most prevalent bacterial family in the protozoa associated bacterial communities. Several taxa not detected or detected in extremely low abundance in the free-living community were enriched in the protozoa associated bacterial community. These include members of the Endomicrobia class, previously identified as protozoa symbionts in the termite gut. Our results show that rumen protozoa harbor prokaryotic communities that are compositionally different from their surroundings, which may be the result of specific tropism between the prokaryotic community and protozoa.

## Introduction

Ciliate protozoa are a diverse group of eukaryotic microorganisms, ubiquitous across many environments and are known to live in close association with their surrounding prokaryotic community (Dziallas et al., 2012; Newbold et al., 2015). The prokaryotic species associated with protozoa were shown to display a wide variety of functions, contributing to the fitness of their hosting cell (Gast et al., 2009). Protozoa associated prokaryotes were shown to serve as electron sinks through nitrogen fixation, acetogenesis or methanogenesis (Yamada et al., 1997; Ohkuma et al., 2015; Tai et al., 2016), provide defense against predators (Vannini et al., 2003; Görtz, 2006; Montagnes et al., 2008) or serve as nutrition for the hosting cell (Gast et al., 2009; Dziallas et al., 2012; Williams and Coleman, 2012). The nature of the microbial association can be ecto- or endosymbiotic, permanent or temporary and obligate or transient (Gast et al., 2009; Newbold et al., 2015) and is thought to benefit both the host (Yamada et al., 1997; Vannini et al., 2003), and their associated symbiotic prokarya (Newbold et al., 2015).

Ciliate protozoa are an integral part of the rumen microbiome and form a large proportion of its microbial biomass (Newbold et al., 2015). In the rumen, protozoa are commonly observed to harbor intracellular and extracellular prokaryotic cells, part of which, thought to be engaged in a symbiotic relationship with the hosting cell (Fenchel and Finlay, 1992; Finlay et al., 1994; Lloyd et al., 1996; Hackstein and Vogels, 1997).

The recurrent observation that elimination of protozoa from the rumen decreases enteric methane emission, has led to an increasing body of research related to protozoa and their relation with methanogenic archaea, the sole methane producers in the rumen (Hook et al., 2010; Malmuthuge and Guan, 2017; Wallace et al., 2017). Several studies have estimated that the relative contribution of protozoa to methane emission in the rumen can reach up to 37% (Finlay et al., 1994; Hook et al., 2010; Morgavi et al., 2010). A recent meta-analysis consolidating 30 years of experimental defaunation procedures, the process of eliminating the protozoa from the rumen, showed an average decrease of 11% in methane emission (Newbold et al., 2015). Additionally, *in vitro* studies demonstrate a close metabolic interaction between methanogens and ciliates, in which mixed or individual species of ciliates incubated with the rumen prokaryotic community lead to an increase in methane production (Newbold et al., 1995; Ushida et al., 1997; Ranilla et al., 2007).

Therefore, exploring the composition of the protozoa associated methanogenic community and their function has become the focus of many studies, attempting to uncover novel mitigation strategies against methane emission (Tapio et al., 2017; Wallace et al., 2017).

Recent studies comparing the free-living methanogenic community in the rumen to the protozoa associated community revealed differences in methanogen composition, with *Methanobrevibacter* as the dominant genus associated with protozoa (Tymensen et al., 2012; Belanche et al., 2014; Wang et al., 2017). The mostly hydrogenotrophic nature of this genus was suggested as the possible reason for its dominance, as rumen protozoa produce a large quantity of H_2_ as part of their metabolism (Paul et al., 1990). In contrast, taxa belonging to the methylotrophic RCC clade of the Thermoplasmata class, recently reclassified as belonging to the Methanomassiliicoccaceae family (Paul et al., 2012; Iino et al., 2013; Whitman et al., 2015), were shown more prominent in the free-living community (Tymensen et al., 2012). Physical interaction between protozoa and prokaryotes was also observed using fluorescent probes targeting the 16S rDNA of archaea and bacteria. Lloyd et al. (1996) observed an extensive presence of extra and intracellular prokaryotic cells across different protozoa species, localized in different compartments within the protozoal cells. This study also pointed out that colonization is not restricted to archaea, and further identified bacterial cells associated with protozoa.

In contrast to the increased focus methanogens received regarding their association with protozoa, the characterization of the bacterial communities associated with the protozoa in the rumen received little attention, and their composition and role, if any, beyond serving as nutrition for the predatory ciliates, remains unclear (Lloyd et al., 1996; Irbis and Ushida, 2004). In this study, we isolated different protozoa size fractions found in the bovine rumen and characterize the ecological features and dynamics of their associated prokaryotic population. We further assessed the differences in identity and composition of the prokaryotic community associated with different protozoa populations.

## Materials and Methods

### Animal Handling and Sampling

The experimental procedures used in this study were approved by the Faculty Animal Policy and Welfare Committee of the Agricultural Research Organization Volcani Research Center approval no. 737/17 IL, in accordance with the guidelines of the Israel Council for Animal Care.

Rumen fluid was sampled from three cows, two of which sampled five times throughout 1 month and one cow sampled twice while the animals were kept under the same diet (Supplementary Table S1). The sampled rumen fluid was passed through eight layers of cheesecloth, and immediately transferred to an oxygen free environment in an anaerobic glove box. In order to obtain different populations of protozoa free of external contamination from the free-living prokaryotic population, the rumen samples underwent a series of size filtration and washings similar to the procedure performed in Belanche et al. (2014). Briefly, the rumen fluid was mixed in a 1:1 ratio with warmed, anaerobic Coleman buffer (Williams and Coleman, 2012), and incubated in a separating funnel for 1 h under anaerobic conditions at 39°C. The settled protozoa fraction was transferred to a fresh tube with warm Coleman buffer and underwent consecutive filtration using nylon net filters (Merck Millipore, Darmstadt, Germany) of different sizes (i.e., 100 μm, 60 μm, 40 μm, 10 μm). The retentate on each filter and the filtrate of the last 10 μm filtering were then washed with warm anaerobic Coleman buffer. Each fraction was washed four times by centrifugation at 500 ×*g* for 5 min and subsequent buffer replacement. The P-<10 fraction had an additional washing step as previous attempts showed that some external contamination remained after four washes. The wash solution from the last washing step was centrifuge at 10,000 ×*g* for 5 min and resuspended in 3 ml of buffer. Direct PCR was performed on the remaining wash solution to confirm the removal of free-living contamination in the filtered samples (Supplementary Figure S1). The free-living fraction was obtained from the whole rumen sample from a separate incubation in Coleman buffer and was centrifuge twice at 500 ×*g* to remove the protozoa fraction from free-living bacteria. Only two-thirds of the supernatant were kept to minimize contamination from the settled protozoa.

### DNA Extraction

DNA extraction was performed as previously described (Stevenson and Weimer, 2007). In brief, cells were lysed by bead disruption using Biospec Mini-Beadbeater-16 (Biospec, Bartlesville, OK, United States) at 3000 RPM for 3 min with phenol followed by phenol/chloroform DNA extraction. The final supernatant was precipitated with 0.6 volume of isopropanol and resuspended overnight in 50–100 μl TE buffer (10 mM Tris-HCl, 1 mM EDTA), then stored at 4°C for short-term use, or archived at -80°C.

### Quantitative Real-Time PCR

Quantitative real-time PCR analysis was performed to quantify the abundance of total bacteria, methanogens, and ciliate protozoa using the standard curve method (Yuan et al., 2006). Briefly, a standard curve was generated for each of the sequences by amplifying serial 10-fold dilutions of gel-extracted PCR products obtained from the amplification of each amplicon. Real-time PCR was performed in a 10 μl reaction mixture containing 5 μl of Absolute Blue SYBR Green Master Mix (Thermo Scientific, Waltham, MA, United States), 0.5 μl of each primer (10 μM working concentration), 3 μl of nuclease-free water and 2 μl of 10 ng DNA templates. Amplification involved one holding cycle at 95°C for 15 min for initial denaturation and activation of the hot-start polymerase system, and then 40 cycles at 95°C for 10 s followed by annealing at 60°C for 15 s and extension at 72°C for 20 s. The primer sequences for the total bacteria were taken from Walter et al. (2000); HDA-forward ACTCCTACGGGAGGCAGCAG and HDA-reverse 5′ GTATTACCGCGGCTGCTGGCAC 3′. 16S rRNA sequences for the methanogens were taken from Zhou et al. (2010); UniMet-forward 5′CCGGAGATGGAACCTGAGAC3′ and UniMet-reverse 5′CGGTCTTGCCCAGCTCTTATTC3′. The 18S rRNA primers sequences for ciliates for real time PCR were the same as the sequences used for sequencing (Tapio et al., 2016).

### Illumina Amplicon Sequencing and Data Analyses

The Research Laboratory Hylab (Rehovot, Israel) performed amplicon sequencing of the ruminal DNA samples as follows: 20 ng of DNA was used in a 25 μl PCR reaction with primers, using PrimeStar Max DNA Polymerase (Takara) for 20 cycles. The PCR reaction was purified using AmpureXP beads, and then a second PCR was performed using the Fluidigm Access Array primers for Illumina to add the adaptor and index sequences. For this reaction 2 μl of the first PCR were amplified in a 10 μl reaction for 10 cycles. The PCR reaction was purified using AmpureXP beads and the concentrations were measured by Qubit. The samples were pooled, run on a DNA D1000 screentape (Agilent) to check for correct size and for the absence of primer-dimers product. The pool was then sequenced on the Illumina MiSeq, using the MiSeq V2-500 cycles sequencing kit. The primers sequences used for the 16S rDNA bacteria and archaea were taken from the updated sequences (2015–present) of the earth microbiome project^1^, amplifying the V4 region 515F (5′-GTGYCAGCMGCCGCGGTAA-3′) and 806R (5′-GGACTACNVGGGTWTCTAAT-3′). Sequencing was performed on a MiSeq platform using the paired end protocol (2 × 250 bp). For the 18S rDNA sequencing, primers specifically designed for ciliates were used from Tapio et al. (2016) with the following sequences: CiliF (5′-CGATGGTAGTGTATTGGAC-3′) and CiliR (5′-GGAGCTGGAATTACCGC-3′). The sequencing data were deposited into the Sequence Read Archive (SRA) of NCBI and can be accessed via accession number SRP156491.

### Data Analysis

Downstream processing of the 16S rDNA data, up to the generation of OTU tables was performed in QIIME v.1.9.1 (Caporaso et al., 2011). After removal of low quality reads below Q24, sequences shorter than 200 bp and chimera removal using Uchime (Edgar et al., 2011), the pick_open_reference_OTU procedure was used with Usearch v.6.1 (Edgar, 2010), with the 97% similarity threshold for OTU clustering. Taxonomic assignment for the bacterial 16S was performed using BLAST against the latest version of the Greengenes database (greengenes_13_8^2^).

After taxonomic assignment, reads belonging to Chloroflexi and Cyanobacteria phyla were further removed from the dataset, as these sequences may be the product of chloroplast amplification commonly found in protozoa and cannot confidently be attributed to the bacterial domain (Huws et al., 2012). For taxonomic assignment of the archaeal sequences, the Rumen and Intestinal Methanogen database (RIM-DB), which enables a deeper classification or rumen methanogenic taxa, was used to categorize the methanogenic OTUs according to defined clades (Seedorf et al., 2014).

After the generation of the OTU table, singletons/doubletons were removed and sub-sampling to even depth of 14,000 reads per sample was performed for all subsequent analyses. Alpha and Beta diversity analyses were performed using the workflow script core_diversity_analysis.py in QIIME. Non-metric multidimensional scaling (NMDS) using the Bray–Curtis dissimilarity metric based on OTU composition (OTU > 97% identity, species level similarity) was used and plotted using the PAleontological STatistics software (PAST) (Hammer et al., 2001). Bonferroni corrected analysis of similarity (ANOSIM) was used to test the significance of the group clustering and Benjamini–Hochberg corrected Student’s *t*-test procedure was used to assess the differences in the mean variability between samples from different fractions across animals and time points. The heatmap and hierarchical clustering of the 16S rDNA relative abundance data was generated with the heatplus package in R (Ploner, 2006). For the heatmap, the data was log_10_ transformed for better visualization. The ciliates 18S rDNA data underwent the same procedures for data analysis except for the similarity threshold for the definition of an OTU, which was set at 100% similarity and taxonomic assignment was performed using the curated database generated by Kittelmann et al. (2015). Statistical analysis of the compositional differences between the fractions was performed using Kruskal–Wallis test, to assess overall significant differences between the fractions. When Kruskal–Wallis indicated a significant difference between the groups, a *post hoc* Wilcoxon rank sum test was performed to determine which paired groups differed from each other. For all the analyses, *P*-values of <0.05 after FDR correction using the Benjamini–Hochberg procedure (Benjamini and Hochberg, 1995) were considered significant.

## Results

### Relative Quantification of the Rumen Archaea, Bacteria, and Ciliates

**FIGURE 1:**
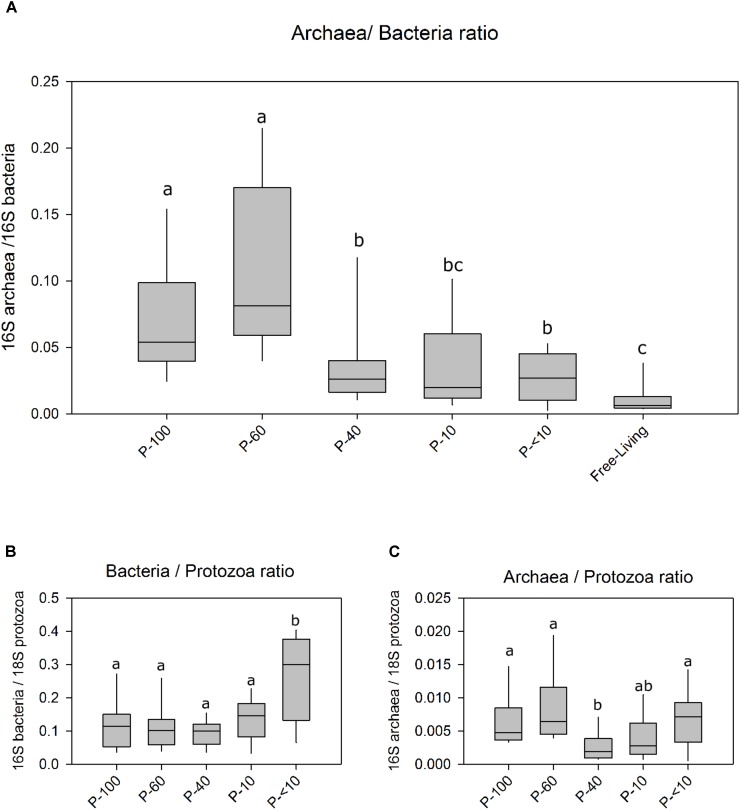
Ratios of bacteria archaea and protozoa across the different fractions using real-time PCR. **(A)** The ratio between archaea 16S and the bacteria 16S in the free living rumen community and each protozoa fraction obtained by size filtering. **(B)** The ratio between bacterial 16S copies and protozoa 18S. **(C)** The ratio between archaea 16S copy number against the protozoa 18S copies. Boxes represent the interquartile range (IQR) between the first and third quartiles (25th and 75th percentiles, respectively) and the horizontal line inside the box defines the median. Whiskers represent the lowest and highest values within 1.5 times the IQR from the first and third quartiles, respectively. Significance was performed using the Wilcoxon rank sum test corrected for multiple comparisons using the Benjamini–Hochberg procedure. The lettering above the boxes indicates the corrected significance between the fractions with boxes not sharing a letter being significantly different at *P* < 0.05.

We quantified the abundance of the bacteria and methanogens and their relative prevalence in association with the different protozoa populations and the free-living community using real time PCR. This analysis revealed that the methanogens/bacteria ratio within the protozoa fractions is significantly higher compared to the ratio found in the free-living fraction (Wilcoxon rank sum, *P* < 0.05; Figure 1A). The communities obtained from the larger filters P-100, P-60 had the highest methanogens proportion relative to bacteria with an average of ∼6% and ∼8%. Fractions P-40, P-10 and P-<10 had a relative proportion of 2.6%, 1.9%, and 2.6% of methanogens/bacteria. The free-living prokaryotic community had 0.8% archaea, relative to bacteria which was significantly lower than the protozoa associated samples (Wilcoxon rank sum, *P* < 0.01; Figure 1). The ratio between bacterial 16S and protozoa 18S did not differ between most of the fractions, and averaged 1.3:10 bacteria/protozoa except for P-<10 which exhibited a significantly higher 1:3 bacteria/protozoa ratio (Figure 1B). 16S methanogen copies were in a significantly higher proportion to 18S protozoa in P-100, P-60 and P-<10 with 1:150, 1:120 and 1:113, respectively, compared to the P-40 and P-10 with a ratio of 1:330 and 1:400 (Wilcoxon rank sum; *P* < 0.01; Figure 1C).

### Ciliate Protozoa Composition Across the Different Size Filters

**FIGURE 2:**
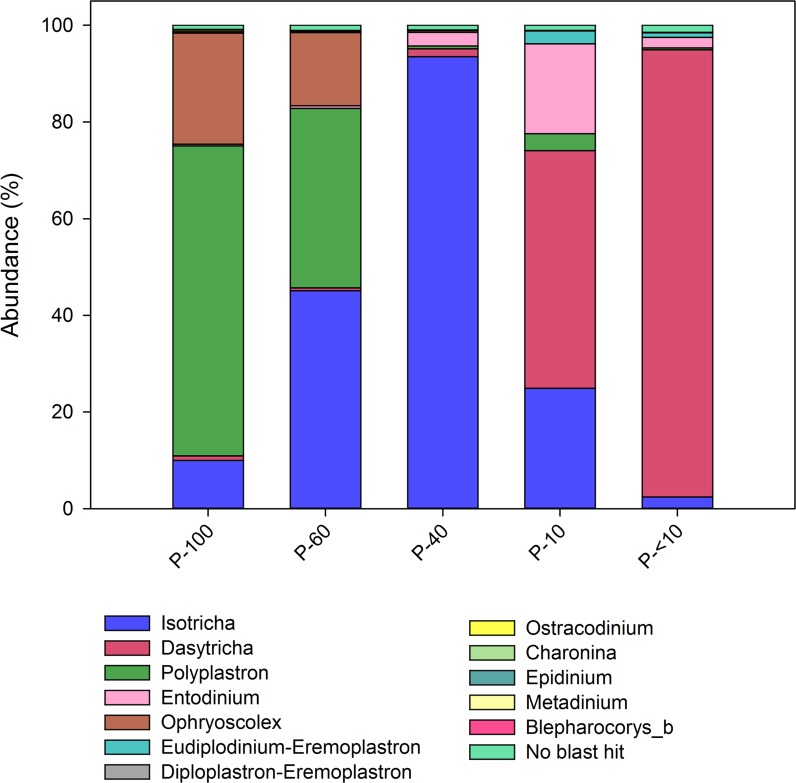
Protozoa distribution obtained from the different filters. Color-coded stack plot showing the genus level protozoa distribution across the different size fractions. Taxonomic assignment was performed using the database obtained from Kittelmann et al. (2015).

We assessed the protozoa composition across the different fractions using 18S amplicon sequencing (Tapio et al., 2016). Quality control and chimera removal using the QIIME pipeline yielded 1,942,623 quality reads, with an average of 30,305 ± 1325 reads per sample. The overall number of OTUs detected by the analysis reached 9,525 based on 100% nt sequence identity between reads. Out of all reads only 1.4% of the reads remained unannotated (Figure 2). In total, we observed 12 protozoa genera across all the samples. *Polyplastron* and *Ophryoscolex* dominated the P-100 and P-60 while P-40 was almost exclusively composed of *Isotricha* (95.5%). The smaller filter P-10 had a mixed population of *Dasytricha, Entodinium* and *Isotricha*, and its filtrate, P-<10 almost exclusively contained members of *Dasytricha* (93.5%; Figure 2). This result shows that using the filtering approach we are able to obtain distinct protozoa populations.

### Amplicon Sequencing of the Prokaryotic 16S rDNA

Sequencing of the 16S rDNA yielded, 3,924,475 quality reads, with an average of 45,354 ± 2748 reads per sample. The overall number of OTUs detected by the analysis reached 12,615 based on ≥97% nt sequence identity between reads. The universal primers used, which capture both bacteria and archaea allowed an assessment of the number of reads within each domain from the same sequencing run. The assignment of the reads to bacteria and archaea revealed that the archaea/bacteria ratio within the protozoa fractions was significantly higher compared to the ratio of the free-living fraction (Wilcoxon rank sum, *P* < 0.05; Supplementary Figure S2). This result validated the qPCR results; however, the proportions obtained from amplicon sequencing were significantly higher compared to real time PCR results (Supplementary Figure S2). The results obtained from the larger size filters P-100, P-60 had, with 10% and 13% of the total prokaryotic community, the highest methanogens proportions. The P-40, P-10, and P-<10 samples had a relative proportion of ∼6%, ∼5%, and ∼4%, respectively. The free-living microbial community had 1.1% of the total prokaryotic community assigned to archaea.

### Structure of the Prokaryotic Community Across Different Rumen Protozoa Fractions

**FIGURE 3:**
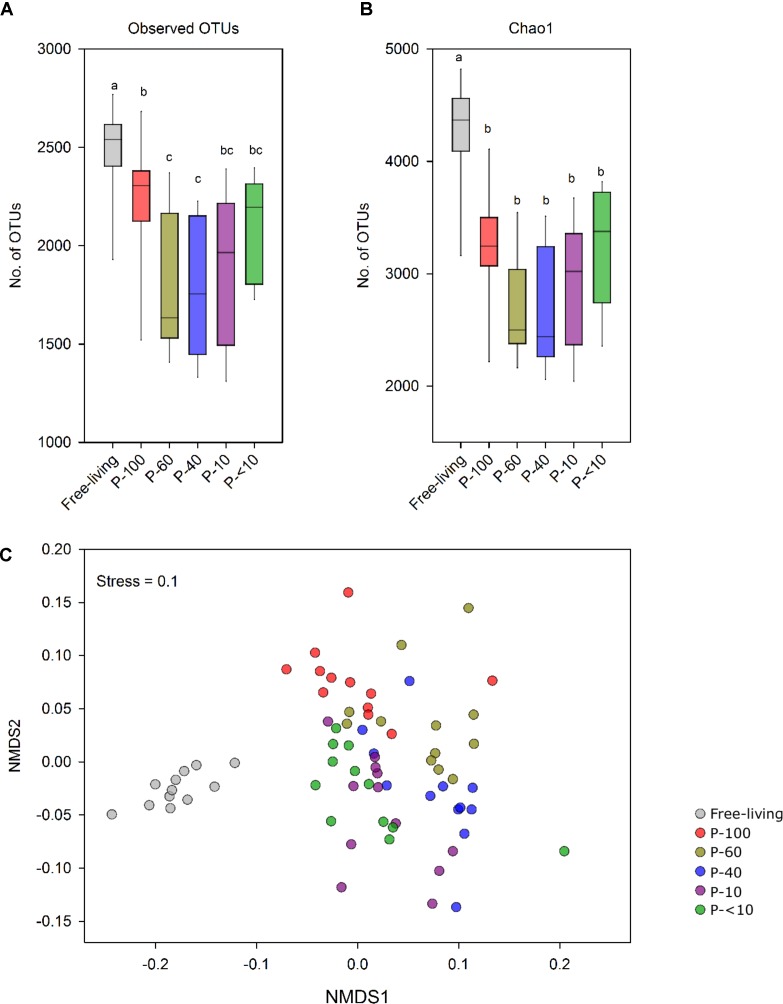
Prokaryotic population structure across the different fractions. **(A)** OTU richness (≥97% similarity) and **(B)** Chao1 index of the free-living and protozoa associated prokaryotic community. **(C)** Non-metric multidimensional scaling (NMDS) based on the OTU similarity matrix obtained using the Bray–Curtis index. Each point represents a different sample plotted according to their OTU composition and abundance. A greater distance between two points infers a lower similarity between the prokaryotic community compositions. Color-coding represents the different fractions assessed.

Alpha diversity analysis showed that the free-living prokaryotic community was significantly richer compared to all protozoa fractions. This was observed by both the number of detected OTUs and the Chao1 index. Furthermore, P-60, P-40, and P-10 had a lower species richness compared to the P-100, P-<10 and free living fractions (*P* < 0.05; Figures 3A,B).

Non-metric multidimensional scaling using the Bray–Curtis dissimilarity metric revealed a distinct clustering between the free-living community and the protozoa associated communities (Figure 3C and Table 1). In addition, a distinct and significant clustering was observed between the different protozoa associated communities obtained from different filter sizes. Notably, the difference between the communities increased with increasing filter size distance (Figure 3C and Table 1). A significant clustering of the samples according to the host animal origin could be observed in the free-living fraction in the cows sampled across five time points (ANOSIM, *R* = 0.68; *P* < 0.01), but no such discrimination could be observed in the protozoa associated prokaryotic communities (Supplementary Figure S3A). The protozoa associated prokaryotic community also exhibited a higher variability, with an average pairwise Bray–Curtis dissimilarity between the different time points of 0.39, 0.42, 0.38, 0.43, and 0.39 for P-100, P-60, P-40, P-10, and P-<10, respectively, compared to the free living community with an average pairwise similarity of 0.32 (Student’s *t*-test; *P* < 0.05; Supplementary Figure S3B).

**Table 1:** Analysis of similarity between the microbial communities in the different fractions.

*P*-value/	Free-living	P-100	P-60	P-40	P-10	P-<10
*R*-value	
Free-living		0.0015	0.0015	0.0015	0.0015	0.0015
P-100	0.5902^∗∗^		0.213	0.003	0.0015	0.0015
P-60	0.6233^∗∗^	0.15		0.1995	0.027	0.0074
P-40	0.5755^∗∗^	0.402^∗∗^	0.1824		1	0.2325
P-10	0.5597^∗∗^	0.3735^∗∗^	0.2158^∗^	0.073		1
P-<10	0.6864^∗∗^	0.4287^∗∗^	0.3125^∗∗^	0.1694	-0.03051	

*This analysis statistically test whether significant differences exists between two or more groups of samples according to the distance obtained from the Bray–Curtis dissimilarity matrix. *R*-value denotes the degree of differences between the groups with *R*-values closer to 1 denoting a highly different community composition. The lower quadrant represent the *R*-values for each group pair and the upper quadrant represents the Bonferroni corrected *P*-value for each pair. ^∗^represents a significance at *P* < 0.05 and ^∗∗^ significance at *P* < 0.01*.

### Comparative Analysis of Protozoa Associated and Free-Living Methanogenic Community

In the free-living community, the Methanomassiliicoccaceae family was the dominant taxon, accounting for an average of 50% of all methanogens reads, followed by the *Methanobrevibacter*, with an average of 34%, although a large variability can be seen within this genus between the different time points and hosts. *Methanomicrobium* had an average of 11% in the free-living community (Supplementary Figure S4). The protozoa associated methanogenic communities were largely dominated by *Methanobrevibacter* (*Mbb.*) with averages ranging between 50% and 80%, followed by the Methanomassiliicoccaceae with averages ranging between 13% and 39% and *Methanomicrobium* ranging between 0.94% and 7.4% across the fractions.

**FIGURE 4:**
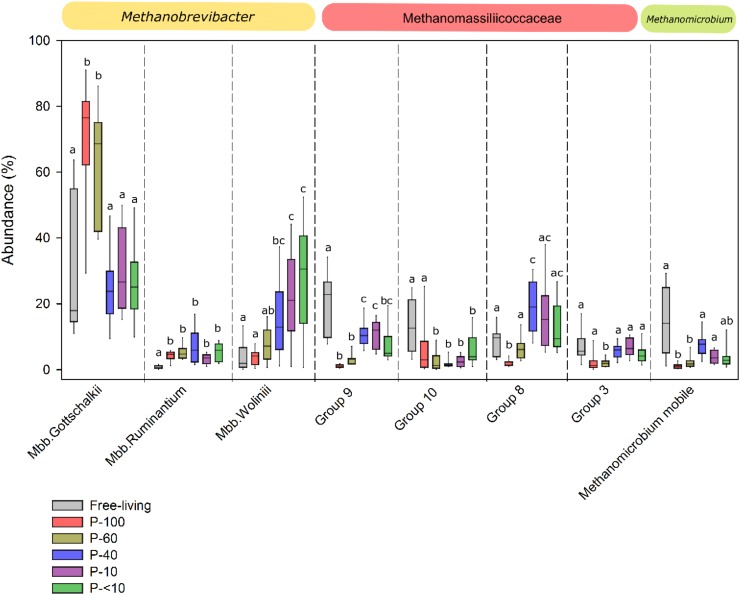
Methanogens composition across the different fractions. Box plot representing the taxonomic distribution methanogens across the different fractions. The taxa are defined according to the lowest taxonomic assignment obtained using RIM-DB database and their corresponding family or genus affiliation is emphasized above each plot. Boxes represent the interquartile range (IQR) between the first and third quartiles (25th and 75th percentiles, respectively) and the horizontal line inside the box defines the median. Whiskers represent the lowest and highest values within 1.5 times the IQR from the first and third quartiles, respectively. The lettering above the boxes denotes the Benjamini–Hochberg corrected significance across the different groups with boxes not sharing a letter being significantly different at *P* < 0.05.

Using RIM-DB, a database specifically oriented toward identification and annotation of rumen methanogenic archaea, we assessed the distribution within the taxonomic groups observed across the different fractions. OTUs that had a high similarity (OTU similarity > 97%) with sequences from this database where thus categorized to several different clades within each of the families or genera observed (Seedorf et al., 2014). *Mbb. gottschalkii, Mbb. wolinii*, and *Mbb. ruminantium* represented the vast majority of OTUs from the *Methanobrevibacter* genus, and exhibited significant differences both between the free-living and protozoa associated population and between the different protozoa fractions (Figure 4). Fractions P-100 and P-60 were characterized by a significantly higher proportion of OTUs associated with *Mbb. gottschalkii* compared to the free-living population and the other protozoa associated fractions (Wilcoxon rank sum, *P* < 0.01; Figure 4). In contrast, OTUs associated with *Mbb. wolinii* were observed in higher proportion in the P-40, P-10, and P-<10 fraction compared to the P-100, P-60 and the free-living fraction (Wilcoxon rank sum, *P* < 0.05). *Mbb. ruminantium* was in a significantly higher proportion in all the protozoa fractions (5.11% average across all protozoa associated fractions) compared to the free-living population (0.83%) (Wilcoxon rank sum, *P* < 0.05).

The Methanomassiliicoccaceae family was composed of several defined sub-groups (Seedorf et al., 2014), which, similarly to *Methanobrevibacter*, had different proportions between the fractions. The proportion of OTUs associated with Group_9 was significantly higher in the free-living fraction, accounting for an average of 21.13% of the total methanogenic OTUs compared to the protozoa associated fractions (Wilcoxon rank sum, *P* < 0.05). In addition, different proportions were observed within this group between the protozoa fractions, with P-100 and P-60 exhibited the lowest proportion with an average of 1.07% and 3.3%, respectively, compared to a significantly higher proportion in fractions P-40, P-10, and P-<10, with 11%, 10.5% and 5.9 %, respectively (Wilcoxon rank sum, *P* < 0.05; Figure 4). The Group_8 clade had a lower proportion in P-100 compared to the free-living fraction, but was higher in the P-40 fraction (*P* < 0.05; Wilcoxon rank sum; Figure 4). *Methanomicrobium mobile* was in a significantly higher proportion in the free-living fraction with an average of 11.86%, compared to P-100 and P-60 protozoa fractions (1% and 2.1%, respectively; *P* < 0.05), and exhibited a non-significant lower proportion in P-40 (8.4%; *P* = 0.29), P-10 (3.8%: *P* = 0.07), and P-<10 (3.6%; *P* = 0.09) fractions (Figure 4).

### Bacterial Distribution Across the Free-Living and Protozoa Fractions

**FIGURE 5:**
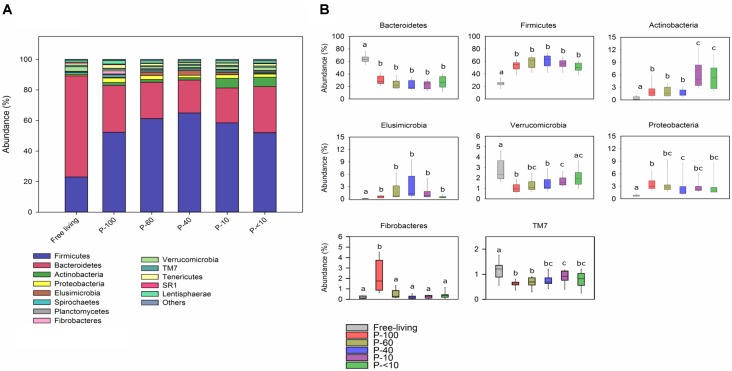
Phylum level bacterial distribution across the fractions. **(A)** Color-coded stack plot showing the average bacterial phylum distribution in the free-living and protozoa associated fractions. **(B)** Box plots showing the relative abundance of the most abundant phyla found across the different fractions. The lettering above the boxes indicates the Benjamini–Hochberg corrected significance across the different groups with boxes not sharing a letter being significantly different at *P* < 0.05.

A total of 46 different bacterial phyla were observed across all samples, among them 14 that had an average relative abundance above 0.5% in at least one of the groups (Figure 5A).

The Bacteroidetes and Firmicutes were the most prevalent phyla within all the samples, which is in agreement with previous rumen microbiome studies (Jami and Mizrahi, 2012; Henderson et al., 2015). However, the Bacteroidetes proportion was significantly higher in the free-living community with an average of 62.8% compared to the protozoa associated communities, in which it had an average of 25% (Wilcoxon rank sum, *P* < 0.001; Figure 5B). In contrast, the Firmicutes was the dominant phylum in all protozoa fractions, accounting for an average of 52–63% compared to a 24% average in the free-living community (Wilcoxon rank sum, *P* < 0.001; across all fractions). Other phyla that had significantly different proportion between the free-living and protozoa fractions include the Actinobacteria, Proteobacteria, and Elusimicrobia which were higher in all of the protozoa associated communities (Figure 5B). In contrast, the Verrucomicrobia had a lower proportion in most of the protozoa associated fractions, compared to the free-living fraction and TM7 was proportionally higher in the free-living population compared to all the protozoa associated fractions (Wilcoxon rank sum, *P* < 0.05). Both Actinobacteria and Fibrobacteres exhibited a significant differential proportion between the protozoa fractions. Fibrobacteres was uniquely enriched in the P-100 fraction (Wilcoxon rank sum, *P* < 0.01; Figure 5B) while Actinobacteria was in a significantly higher proportion in P-10 and P-<10 compared to all other fractions with averages of 5.8% and 6%, respectively (Wilcoxon rank sum, *P* < 0.01).

**FIGURE 6:**
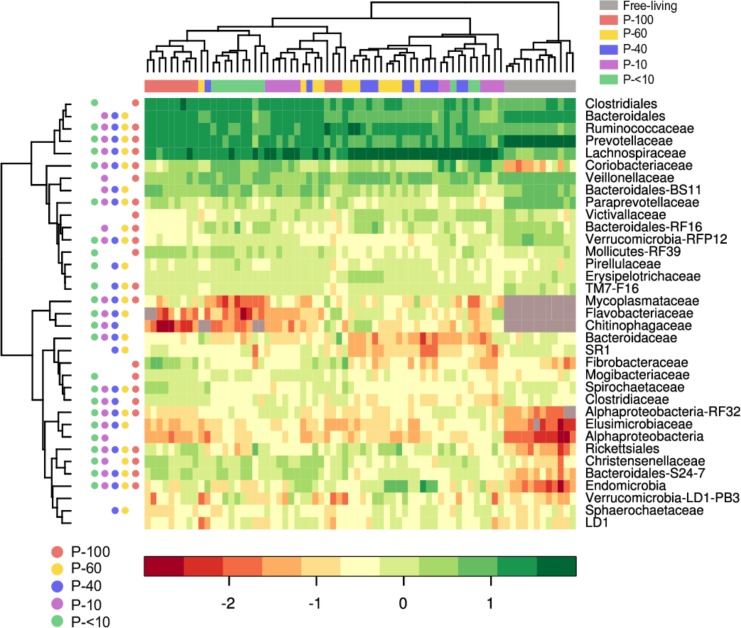
Heat map and hierarchical clustering of the family-level bacterial composition across the different fractions. The log-transformed abundance data of the 35 most abundant family level taxa in the different fractions is depicted. Each vertical lane corresponds to a sample and the different colors below the horizontal dendrogram denotes sample provenance across the different fractions. The filled circles next to the vertical dendrogram represent the FDR corrected significance between the free-living and each individual protozoa associated community groups (Wilcoxon rank sum, *P* < 0.05, for each row). For example, the Clostridiales in the free-living community is in a significantly different proportion compared to P-100, and P-<10 but not P-60, P-40, and P-10. The grey squares inside the heat map denotes taxa that were not detected in a sample. Taxa that could not be assigned to a family are displayed using the lowest taxonomic level that could be assigned to them.

We further depicted the 35 most abundant families out of a total of 315 families that had a mean relative abundance of at least 0.5% in at least one of the fractions (Figure 6).

The higher proportions observed within the Firmicutes phylum in the protozoa associated fractions was mostly driven by the presence of the Lachnospiraceae family. This family appeared in a significantly higher proportion in all of the protozoa associated communities with an average of 12.7%, 25.4%, 32%, 28.9%, and 24% for the P-100, P-60, P-40, P-10, and P-<10 fractions, respectively, compared to an 3.75% on average in the free-living community (Wilcoxon rank sum; *P* < 0.01, Figure 6).

**FIGURE 7:**
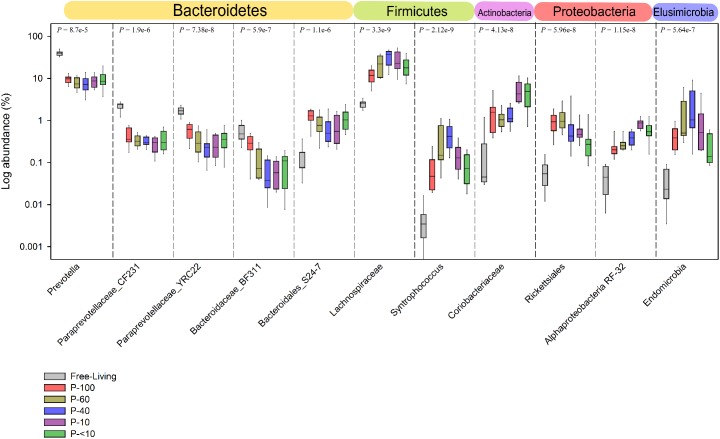
Taxa exhibiting the highest difference between the free-living and protozoa associated communities. Taxa which exhibited the largest difference in relative abundance between the free-living and protozoa associated communities. The taxa shown are represented at the lowest taxonomic affiliation that could be assigned to them up to the genus level. The *P*-values above each separate plot were generated using the Kruskal–Wallis test, corrected for multiple comparisons using the Benjamini–Hochberg procedure.

Prevotellaceae and its most abundant genus in the rumen, the *Prevotella*, was in a significantly lower proportion compared to the free-living across all the protozoa associated communities (*P* < 0.001; Figures 6, 7 and Supplementary Table S2). Other significantly and consistently underrepresented taxa in the protozoa associated communities included undefined genera of Paraprevotellaceae, with 4.5% free-living vs. ∼0.8% protozoa associated communities (Wilcoxon rank sum; *P* < 0.01, Figure 7 and Supplementary Table S2) and Bacteroidaceae family BF311 (Wilcoxon rank sum; *P* < 0.01, Figure 7 and Supplementary Table S2), both belonging to the Bacteroidetes phylum (Figure 7 and Supplementary Table S2). From the Bacteroidetes phylum, the Bacteroidales S24-7 family was in a significantly higher proportion in the protozoa associated communities compared to the free-living community (Wilcoxon rank sum, *P* < 0.001; Figure 6). Within the Actinobacteria phylum, a significantly higher proportion of Coriobacteriaceae was observed in the protozoa associated bacterial community (Figures 6, 7). This family was significantly more abundant within P-10 and P-<10 compared to the other protozoa fractions, with averages of 5.6% and 5.2%, respectively (Wilcoxon rank sum, *P* < 0.01; Figure 7 and Supplementary Table S2). As observed in the phylum distribution, the Fibrobacter genus was uniquely enriched in the P-100 fraction compared to all other protozoa associated free living communities (Wilcoxon rank sum, *P* < 0.01; Figure 6 and Supplementary Table S2). We additionally identified a cluster of several taxa which were seen either in low proportion or not detected in the free-living community but enriched in the protozoa associated communities. These included the Chitinophagaceae of the class Sphingobacteria, Flavobacteriaceae (Flavobacteriia), Mycoplasmataceae (Mollicutes) and members of the Alpha-proteobacteria class including members of the Rickettsiales order (Figures 6, 7 and Supplementary Table S2). Within those less abundant phyla, we observed a significantly higher proportion of Endomicrobia across all protozoa associated communities reaching up to 10% of the bacterial communities in the P-60 and P-40 fractions (Figure 7 and Supplementary Table S2). Notably, the Endomicrobia is a known endosymbiont of flagellate protozoa within the gut of termites, where the flagellates is an obligatory symbiont and has crucial role in cellulose digestion (Honigberg, 1970; Inoue et al., 1997).

## Discussion

The goal of this study was to identify and characterize the prokaryotic communities associated with different ciliate populations found in the bovine rumen and assess whether their composition differs from the free-living communities. Both sequencing and real time PCR results revealed a higher methanogens/bacteria ratio in the samples associated with the different protozoa populations, strengthening the mounting evidence that a strong functional association exists between rumen ciliates and methanogens (Finlay et al., 1994; Ushida et al., 1997; Newbold et al., 2015). Interestingly a higher proportion of archaea/bacteria ratio was detected within the larger protozoa species, suggesting a differential tropism toward the methanogenic population between different protozoa species in the rumen. This is in contrast to Belanche et al. (2014), which did not observe different proportions of methanogens in the communities associated with different protozoa populations in sheep. The discrepancy seen may be related to differences in methodology, such as the different animals used for the experiment, different animal management, diet and sampling time, or the primers used for the detection of methanogens. The latter option may also be responsible for the different results obtained from real-time PCR quantification and the sequencing results in the current study, as it was previously shown that different primer usage carries significant biases when quantifying and characterizing rumen methanogenic population (Tymensen and McAllister, 2012).

Analysis of the prokaryotic OTUs ecological features (i.e., Alpha and Beta-diversity) revealed significant differences in the overall structure of the prokaryotic communities associated with protozoa compared to the free-living community. These results are in agreement with the previously shown differences in methanogenic population structure associated with protozoa compared to the free-living population in the rumen, and suggest that these compositional differences extend to the whole prokaryotic community (Tymensen and McAllister, 2012; Tymensen et al., 2012; Belanche et al., 2014). In addition to the observed differences between the free-living and protozoa associated prokaryotic community, each protozoa fraction was also characterized by a different prokaryotic composition, as the prokaryotic communities obtained from different size filters also clustered apart from one another. This suggests that the association between protozoa species and their surrounding prokaryotic cells may involve mechanisms for selective interactions.

In line with previous observations, the *Methanobrevibacter* was the dominant genus in most of the protozoa fractions (Tymensen et al., 2012). This recurring observation of *Methanobrevibacter*/protozoa association, a genus mainly associated with hydrogenotrophic activity, gives further support the notion that methanogen/protozoa association in the rumen is likely the consequences of the protozoa high hydrogen production capacities (Finlay et al., 1994; Ushida et al., 1997; Tymensen et al., 2012; Newbold et al., 2015).

We also observed significantly different proportions of *Mbb. gottschalkii and Mbb. Wolinii* between the protozoa fractions. Several studies linked high methane emission in cattle with the higher presence of *Mbb. gottschalkii* and other members of the SGMT (*Mbb. smithii, Mbb.* *gottschalkii, Mbb. millerae*, and *Mbb. thaueri)* methanogenic group in the rumen (Danielsson et al., 2012, 2017). This observation, coupled with the differential proportions of this species between the different protozoa populations suggest that a subset of protozoa may have a greater involvement in enhancing methane production. Indeed, this possibility was previously investigated in a study by Belanche et al. (2015), which showed that holotrich protozoa had a greater contribution to methane emission in sheep. Similarly to the *Methanobrevibacter* genus, specific clades belonging to the Methanomassiliicoccaceae family, exhibited differential proportions between the different protozoa associated communities. However, these clade assignments remain limited to the phylogenetic analysis of mostly uncultured species, and no additional information is available on the specific genomic features of these groups and their role in the rumen ecosystem (Seedorf et al., 2014).

So far, only a limited number of studies focused on characterizing the bacterial community associated with rumen ciliate protozoa (Lloyd et al., 1996; Irbis and Ushida, 2004; Boeckaert et al., 2009; Wang et al., 2017). Our analysis revealed broad compositional differences between the free-living bacterial community and the protozoa associated community. These can already be observed at the phylum level where the proportions of several phyla, such as the Firmicutes and Proteobacteria were significantly higher in the protozoa associated communities. Preferential association between protozoa and specific phyla was observed in a recent study assessing the phylogenetic affiliation of bacteria associated with marine and freshwater ciliates (Gong et al., 2016). In this study, the authors showed that taxa belonging to proteobacteria were highly enriched in protozoa. The authors proposed that this selective association is related to two protein secretion systems, more prominent in Gamma- and Alpha-proteobacteria, which may have a significant role in promoting marine protozoa–bacteria associations. Thus, specific genomic features, more common to specific bacterial phyla, may be responsible for the selective association with protozoa. Such genomic features were also observed, in the context of methanogens/protozoa association in the rumen (Ng et al., 2016). A recent study revealed that the genomes of several *Methanobrevibacter* species, such as *Mbb. ruminantium*, contain adhesin-like proteins with high affinity to several protozoa species (Ng et al., 2016), which may explain the observed higher tropism of this genus toward association with protozoa.

The Lachnospiraceae and Ruminococcaceae families were highly enriched in the protozoa associated bacterial communities compared to the free-living community.

The identity and role of most members of Lachnospiraceae and Ruminococcaceae in the rumen remains unclear, however, several recent studies assessing reductive acetogenesis in the rumen uncovered that both Lachnospiraceae and Ruminococcaceae were prominent in culture media enriching for homoacetogenic functions (Gagen et al., 2010, 2014, 2015; Yang et al., 2016). Similarly to methanogens, bacteria capable of performing reductive acetogenesis from CO_2_ and H_2_ as an electron donor to produce acetate, may find the protozoa local environment advantageous. Homoacetogens are thought to be rare in the rumen as they are outcompeted by the methanogens (Cord-Ruwisch et al., 1988; Morvan et al., 1994). However, acetogens and other hydrogen-utilizing bacteria can be observed colonizing the rumen of newborn animals and are abundant during initial rumen development (Fonty et al., 1987, 2007; Morvan et al., 1994; Friedman et al., 2017). Strengthening the possibility of prevalence of acetogens in the protozoa associated communities is the enrichment of taxa assigned to the genera *Syntrophococcus* (OTU similarity > 93.8% with the closest *Syntrophococcus sucromutans* isolate) and *Blautia* (OTU similarity > 95%). *S. sucromutans*, the only cultivated representative of the *Syntrophococcus* genus, is a known rumen acetogen while *Blautia* has several members known to reside in the rumen identified as acetogenic (Krumholz and Bryant, 1986; Doré and Bryant, 1990; Bernalier et al., 1996; Schiel-Bengelsdorf and Dürre, 2012). Our results thus suggest that the protozoa may not only be an attractive environment for hydrogenotrophic methanogens species but also harbor a wider array of bacteria capable of various hydrogenotrophic functions. Such types of interactions are not unprecedented and were recently proposed in protozoa in the gut of wood feeding termites. In the termite gut, the genome of one protozoa associated symbiont was shown to harbor the Wood–Ljungdahl pathway while another symbiont encodes for genes related to sulfate/fumarate reduction, another hydrogenotrophic activity found in the rumen (Ikeda-Ohtsubo et al., 2016; Kuwahara et al., 2017).

In addition to the differential abundance of shared taxa between the free-living and protozoa associated fraction. We observed several bacterial taxa not detected or appearing in low abundance in the free-living samples and enriched in the protozoa associated fractions. These included Proteobacterial lineages such as Rickettsiales and other undefined Alpha-proteobacteria. Irbis and Ushida (2004), made a similar observation in a study in which protozoa incubated for 48 h with antibiotics, led to the enrichment of several Proteobacteria species, one of which previously identified as a known symbiont of the freshwater ciliate *Euplotes aediculatus*. Other bacterial taxa exclusively seen in the protozoa community include members *Sphingobacteria*, and *Flavobacterium*. Interestingly, members of these taxa have been previously shown to be engaged in a symbiotic relationship with the ecto-parasitic ciliate *Ichthyophthirius multifiliis* (Sun et al., 2009).

Our analyses revealed the high prevalence of OTUs belonging to the Endomicrobia class, which could reach up to 10% of the protozoa associated bacterial communities. Several studies identified members of this class engaged in an obligate endosymbiotic relationship with flagellate protozoa species in the wood feeding termites (Ikeda-Ohtsubo et al., 2010; Zheng and Brune, 2015; Mikaelyan et al., 2017). Notably, Endomicrobia are occasionally seen in deep sequencing efforts characterizing the microbiome of foregut and hindgut animals (Hook et al., 2011; Jami et al., 2013; Mach et al., 2017). However, the limited information on these taxa and the fact that it usually represent a low proportion of the observed diversity in the rumen, usually results in this phylum being largely ignored. Ikeda-Ohtsubo et al. (2010) suggested, based on the recurrence of this lineage across different gut systems, including the rumen, that they may represent non-symbiotic species, phylogenetically related to those found in termite protozoa. However, the high proportion observed in the protozoa associated bacterial communities suggests that the association between Endomicrobia and protozoa is not limited to the termite gut and may be widespread in different environments, including the rumen.

## Conclusion

The identity and role of prokaryotic microorganisms associated with protozoa remains largely unclear. To our knowledge this is the first comprehensive study of the overall prokaryotic population found associated with different ciliate protozoa populations, aimed at characterizing the structure of both bacterial and archaeal taxa. The protozoa feed on the prokaryotic members of the microbiome and therefore it is possible that a part of the prokaryotic populations observed serve this purpose (Williams and Coleman, 2012). The difficulty to obtain pure cultures of protozoa hinders the possibility to study specific interactions with the associated prokaryotic species.

The specificity of each prokaryotic community across different protozoa fraction isolated, the proportional dominance of several taxa, and the observed enrichment of bacteria previously linked to protozoa symbiosis in other environments, including the cellulose degrading termite gut system, suggest that the prokaryotic community may have a wide variety of functional interactions, conserved across different environments, with their neighboring ciliate population.

## Author Contributions

EJ and BL conceived and performed the experiments and analyzed the data. EJ wrote the manuscript.

## Conflict of Interest Statement

The authors declare that the research was conducted in the absence of any commercial or financial relationships that could be construed as a potential conflict of interest.

## Footnotes

1 http://www.earthmicrobiome.org/

2 http://greengenes.lbl.gov
