# Supplementary material for: Exploring the Prokaryotic Community Associated With the Rumen Ciliate Protozoa Population
Source: Front Microbiol. 2018 Oct 29;9:2526. doi: 10.3389/fmicb.2018.02526 (PMC6217230; doi:10.3389/fmicb.2018.02526)
Supplement: Supplementary file 1 [file Data_Sheet_1.PDF]

Table S1. Formulated ingredients (g/kg dry matter) of the standard diets used in the experiment.

Feed component	Concentration (g/kg dry matter)
Wheat silage	100
Oat hay	80
Corn silage	100
Clover hay	23
Soybean hulls	78
Soybean meal (solvent-extracted)	22
Ground corn grain	129
Ground barley grain	87
Ground wheat grain	44
Whole cotton seeds	20
Corn gluten feed	96
Corn distillers dry grain	89
Rapeseed meal	38
Whey solids	36
$NaHCO_3$	7.4
NaCl	6
$CaCO_3$	9
Ca-LCFA[a]	14
Soy Molasses	17
Urea	4
Trace mineral + vitamin mixture[b]	0.6

[a] The trace mineral + vitamin mix contained (g/kg DM): Zn, 24; Fe, 24; Cu, D_3, 3,200,000 IU; Vit. E, 48,000 IU.

[b] Calcium salts of long-chain fatty acids.
