# Supplementary material for: Exploring the Prokaryotic Community Associated With the Rumen Ciliate Protozoa Population
Source: Front Microbiol. 2018 Oct 29;9:2526. doi: 10.3389/fmicb.2018.02526 (PMC6217230; doi:10.3389/fmicb.2018.02526)
Supplement: Supplementary file 2 [file Data_Sheet_2.PDF]

Table S2. Genus level taxa distribution. Genus level distribution across the different fractions of the most abundant (relative abundance > 0.5%, in at least one group). Pairwise significance was obtained using Wilcoxon rank sum test was used corrected using the Benjamini-Hochberg procedure. Superscript letters denote the significance between the groups in each row, with values not sharing a letter being significant at $P < 0.05$.

Taxa \ fraction	Free-living	P-100	P-60	P-40	P-10	P-<10
Actinobacteria						
Coriobacteriaceae	0.21[a]	1.12[b]	1.12[b]	1.22[b]	5.14[c]	6.255[c]
Bacteroidetes						
Bacteroidales	12.62[a]	12.24[ab]	7.29[abc]	6.51[bc]	7.22[bc]	10.03[abc]
Segetibacter	0.0001	0.25	0.39	0.54	0.09	0.045
Paraprevotellaceae	4.17[a]	0.89[b]	0.42[b]	0.36[b]	0.25[b]	0.66[b]
Bacteroidaceae BF311	0.52[a]	0.27[a]	0.09[b]	0.06[b]	0.06[b]	0.08[b]
Bacteroidales BS11	3.17[a]	2.49[ab]	1.57[bc]	1.51[c]	1.79[bc]	2.94[a]
Prevotella	39.42	8.51[b]	7.25[b]	7.67[b]	7.73[b]	9.23[b]
Bacteroidales RF16	1.63[a]	0.35[b]	0.74[b]	1.17[ab]	0.77[b]	1.15[ab]
Bacteroidales S24-7	0.095[a]	1.24[b]	0.82[b]	0.70[b]	0.84[b]	1.30[b]
Capnocytophaga	<0.001[a]	0.073[b]	0.31[bc]	0.31[c]	0.13[b]	0.073[b]
Elusimicrobia						
Endomicrobia	0.03[a]	0.48[b]	1.96[b]	2.52[b]	1.22[b]	0.22[b]
Fibrobacteres						
Fibrobacter	0.23[a]	2.3[b]	0.48[a]	0.2[a]	0.23[a]	0.3[a]
Firmicutes						
Clostridiales	7.68[a]	15.42[b]	9.51[c]	8.43[ac]	10.59[ac]	12.35[bc]
Mogibacteriaceae	0.22[a]	0.45[bc]	0.27[ac]	0.27[a]	0.29[ac]	0.39[bc]
Christensenellaceae	0.25[a]	1.21[b]	0.74[bc]	0.60[ac]	0.81[bc]	1.09[bc]
Lachnospiraceae	2.38[a]	10.04[b]	21.08[c]	29.9[c]	27.05[c]	21.69[bc]
Butyrivibrio	0.52[a]	0.87[b]	0.77[a]	0.67[a]	1.05[a]	1.05[b]
Syntrophococcus	0.0034[a]	0.08[b]	0.43[bc]	0.5[c]	0.14[bc]	0.09[b]
Ruminococcaceae	3.83[a]	9.35[b]	10.27[b]	9.34[b]	5.01[c]	5.23[c]
Clostridium	0.001[a]	0.03[b]	0.02[b]	0.02[b]	0.55[c]	0.51[c]
Ruminococcus	1.01[a]	3.69[b]	2.44[b]	1.91[a]	2.56[b]	1.27[a]
Veillonellaceae	1.44[a]	0.63[b]	1.49[a]	2.17[a]	0.81[b]	0.90[ab]
Selenomonas	0.44[a]	0.16[a]	0.28[a]	0.52[a]	0.20[ab]	0.16[b]
Succiniclasticum	4.00 [a]	1.40[b]	1.48[b]	1.65[b]	1.26[b]	2.69[a]
LD1	0.25	0.25	0.39	0.49	0.40	0.36
Proteobacteria						
Alphaproteobacteria RF32	0.040[a]	0.14[b]	0.22[b]	0.26[b]	0.68[c]	0.50[c]
Rickettsiales	0.07[a]	1.0[b]	1.32[b]	0.53[b]	0.56[bc]	0.32[c]
Spirochaetes						
Treponema	0.68[a]	1.07[b]	0.30[c]	0.32[c]	0.17[c]	0.09[d]
Tenericutes						
Mycoplasmataceae	0.0	0.55	0.50	0.15	0.36	0.03
Mollicutes RF39	0.93[a]	1.41[b]	0.59[a]	0.56[a]	0.71[a]	1.31[b]
TM7	1.11[a]	0.52[b]	0.54[b]	0.68[b]	0.81[ac]	0.77[bc]
Verrucomicrobia						
Verruco-5 RFP12 (family)	2.28[a]	0.36[b]	0.31[b]	0.35[b]	0.73[c]	0.87[c]
