# Supplementary material for: Exploring the Prokaryotic Community Associated With the Rumen Ciliate Protozoa Population
Source: Front Microbiol. 2018 Oct 29;9:2526. doi: 10.3389/fmicb.2018.02526 (PMC6217230; doi:10.3389/fmicb.2018.02526)

Figure S1. Agarose gel electrophoresis of the pooled bacterial 16S PCR product of the washing buffer and protozoa pellet from each filtration procedures. Lanes 1-5 represent the PCR product the washing buffer used for the last cleaning step. The lack of product in these lanes signify the successful removal of external contaminations stemming from the free-living population in the protozoa fractions isolated. Lanes 6-10 represent the bacterial 16S PCR product of the protozoal pellet obtained showing the presence of bacteria. Lanes N and P represents the negative and positive control (bacterial extract from the rumen) respectively. Lane L is the 1kb ladder used showing the correct ~200bp PCR product size from the bacterial 16S rDNA amplification.

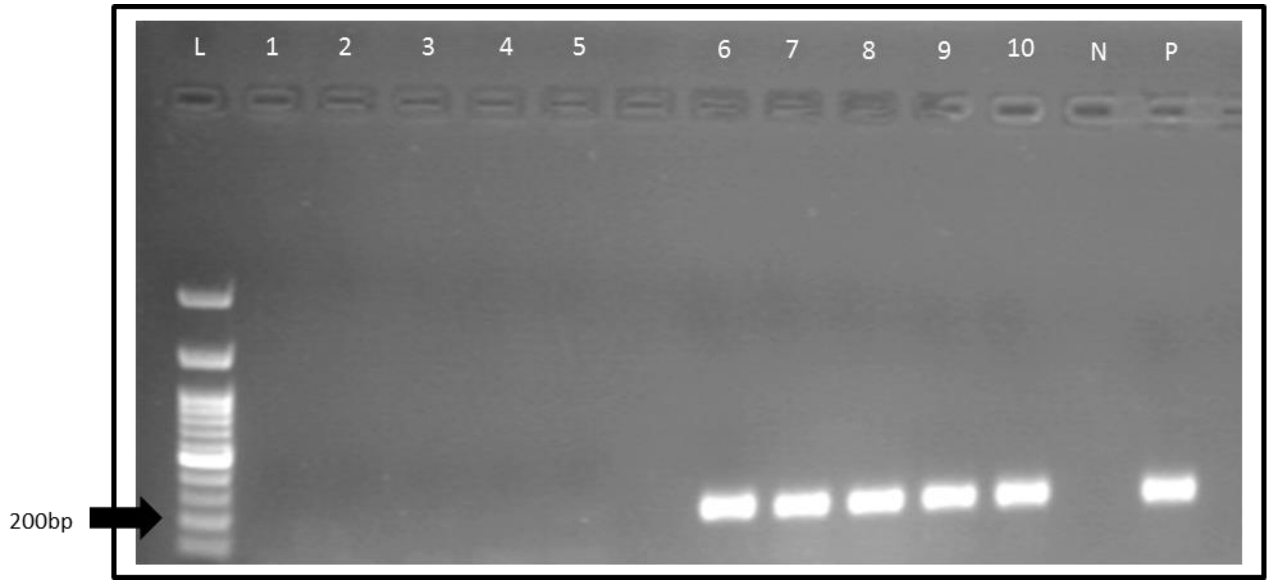

Fig S2. Comparative analysis of the archaea/bacteria ratio obtained between rt-PCR and amplicon sequencing. Each box represent the ratio between archaea and bacteria across the different fractions as observed by sequencing (dark grey boxes) and real time PCR (light grey boxes). Boxes represent the interquartile range (IQR) between the first and third quartiles (25th and 75th percentiles, respectively) and the horizontal line inside the box defines the median. Whiskers represent the lowest and highest values within 1.5 times the IQR from the first and third quartiles, respectively. Significance was performed using the Wilcoxon rank sum test corrected for multiple comparisons using the Benjamini–Hochberg procedure between the sequencing and real time PCR results for each fraction. The asterisk above the boxes denotes that a significance of $P < 0.05$ between the sequencing and rt-PCR values for each fraction. N.S above the boxes denotes that no significance was found between sequencing and rt-PCR.

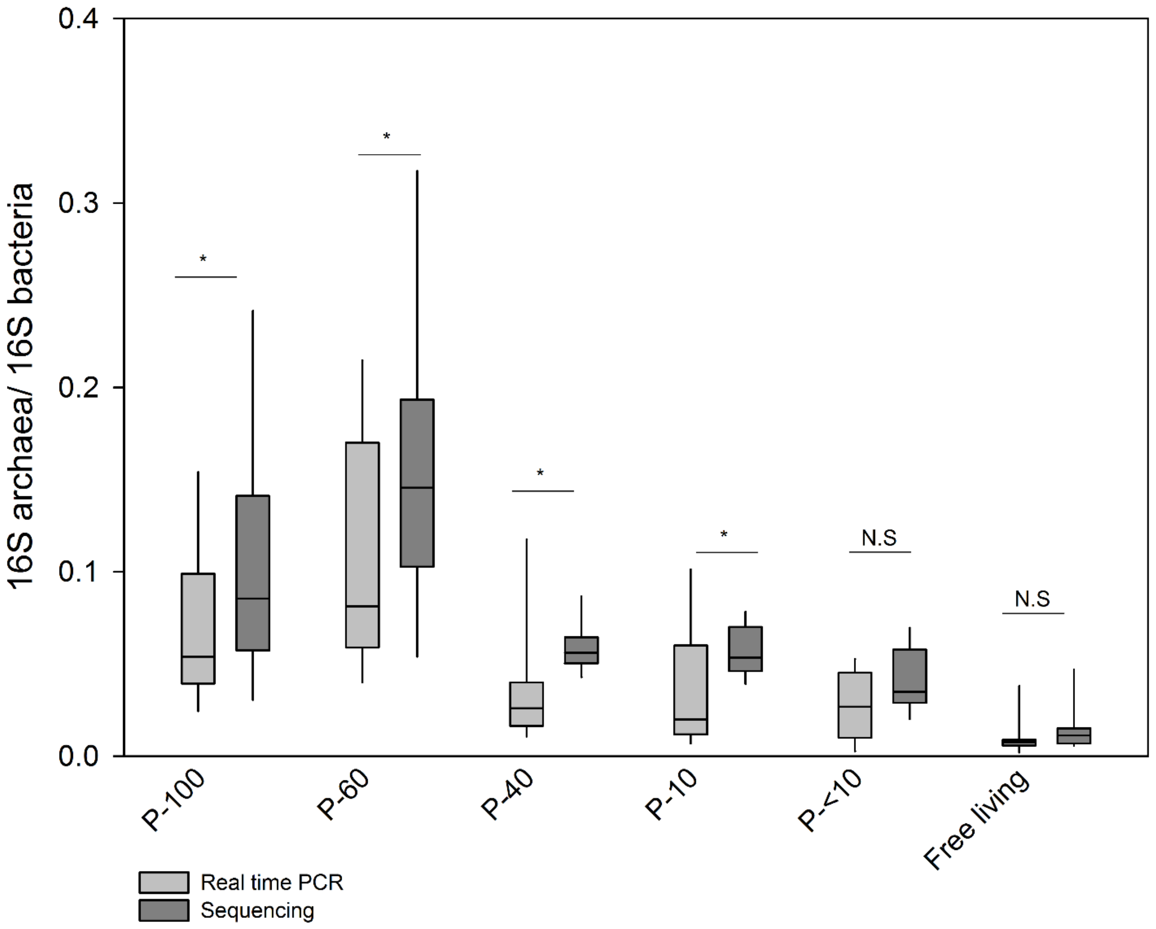

Figure S3. Beta diversity across time and animal sampled. (a) NMDS clustering based on the Bray-Curtis distance according to fraction and host animal. C1 and C2 refers to the two cows sampled throughout the experiments. The convex hulls were added for visualization of clustering. The table below the plot displays the ANOSIM R-values, and the Bonferroni corrected P-values between each animal for each fraction. **(b)** Bar plot showing the average pairwise Bray-Curtis dissimilarity across the different sampling time points within each fraction for each cow sampled. The lettering above the bars indicates the Benjamini–Hochberg corrected significance across the different groups with groups not sharing a letter being considered significantly different at $P < 0.05$.

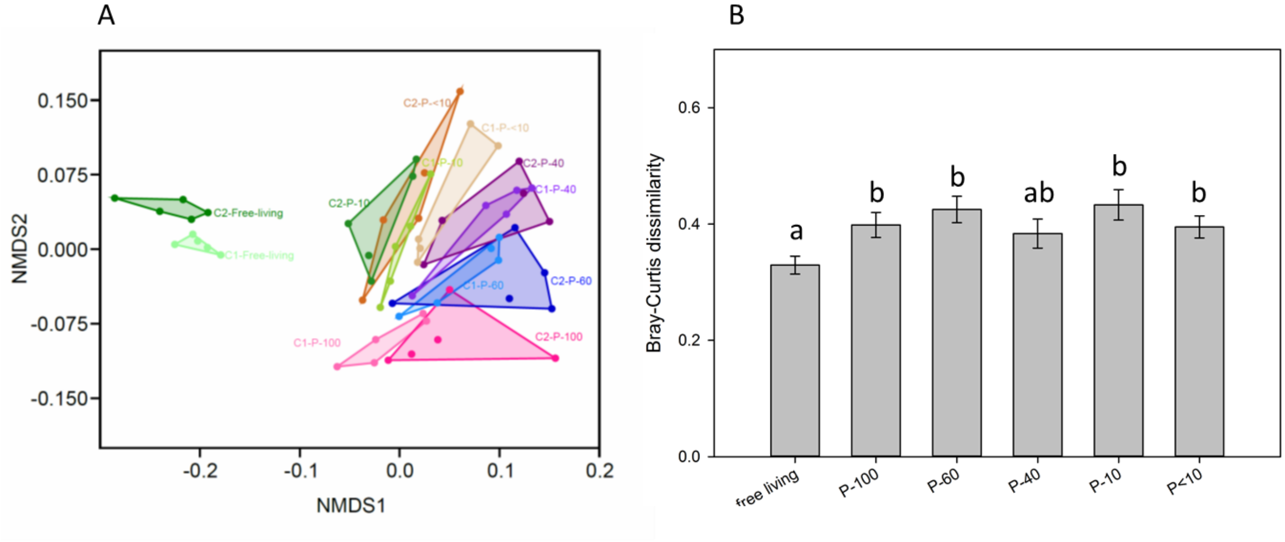

ANOSIM	Free-living	P-100	P-60	P-40	P-10	P<10
R-value	0.682	0.328	0.234	0.18	0.104	0.128
P-value	0.0083	0.094	0.28	0.54	0.52	0.41

Figure S4. Relative abundance of the three major methanogenic taxa across the different fractions. Box plot showing three main methanogenic taxa in the free living and protozoa. The taxa are defined according to the lowest taxonomic assignment available and their corresponding class or order affiliation is emphasized in parenthesis. The lettering above the boxes denotes the Benjamini–Hochberg corrected significance across the different groups with boxes not sharing a letter being significantly different at P < 0.05.

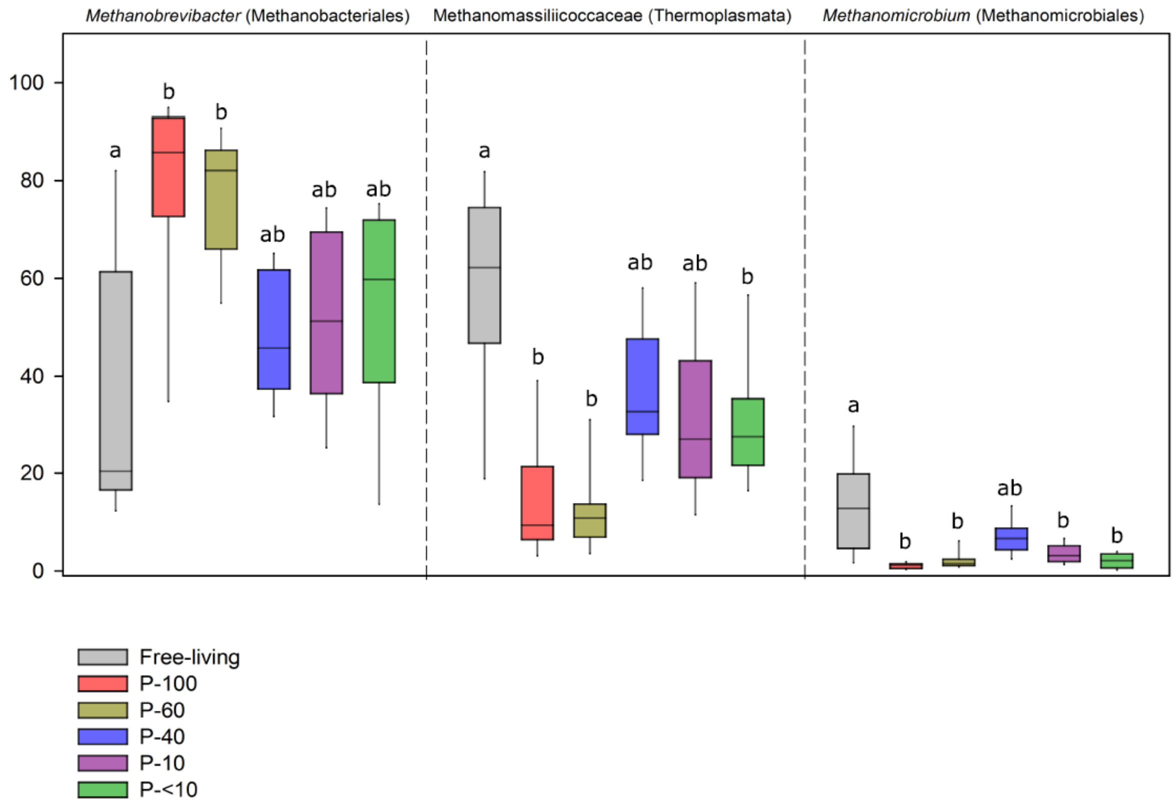

Supplement: Supplementary file 3 [file Presentation_1.PDF]
